# Validation and use of hair cortisol as a measure of chronic stress in eastern chipmunks (*Tamias striatus*)

**DOI:** 10.1093/conphys/cou055

**Published:** 2014-12-05

**Authors:** Gabriela F. Mastromonaco, Kelsey Gunn, H. McCurdy-Adams, D. B. Edwards, Albrecht I. Schulte-Hostedde

**Affiliations:** 1 Reproductive Physiology, Toronto Zoo, 361A Old Finch Avenue, Toronto, Ontario, CanadaM1B 5K7; 2 Department of Biology, Laurentian University, 935 Ramsey Lake Road, Sudbury, Ontario, CanadaP3E 2C6

**Keywords:** ACTH challenge, faecal cortisol, logging

## Abstract

Stress levels of individuals are documented using glucocorticoid concentrations (including cortisol) in blood, saliva, urine or faeces, which provide information about stress hormones during a short period of time (minutes to days). In mammals, use of hair cortisol analysis allows for the assessment of prolonged stress over weeks and months and provides information on chronic stress levels without bias associated with handling. Here, we validate hair cortisol analysis in wild rodents using exogenous adrenocorticotrophic hormone (ACTH challenge) and apply the technique to evaluate stress in eastern chipmunks inhabiting logged and natural sites. Chipmunks were subjected to a mark–recapture study and injected weekly with ACTH (Synacthen Depot) or saline, with hair being collected at the conclusion of the challenge. Subsequently, faecal and hair samples were collected from chipmunks occupying logged and natural sites to assess the utility of hair cortisol in comparison with faecal cortisol metabolites. Following extraction, cortisol concentrations were quantified in hair and faecal extracts by enzyme immunoassay. Hair cortisol concentrations were significantly elevated in samples from ACTH-injected chipmunks compared with saline-injected control animals (five times higher). Chipmunks inhabiting logged sites had increased faecal cortisol metabolite concentrations compared with those in natural sites, but no differences were observed in hair cortisol concentrations. Faecal cortisol metabolite levels were positively correlated with hair cortisol levels in chipmunks. Hair cortisol levels reflect changes in circulating cortisol levels and can be used to evaluate the adrenal stress response, and thus stress, in natural populations. Nonetheless, because of the differences in the temporal scale of stress that hair and faeces represent, we caution the use of hair cortisol for detecting differences in physiological stress when comparing individuals within populations and suggest that it is best suited to examining population-level differences.

## Abstract (precis)

Cortisol is a hormone released when animals experience stress. We validated the measurement of cortisol from hair for use in wildlife using wild chipmunks, and tested the use of hair cortisol by measuring cortisol from chipmunks captured in natural and logged sites.

## Introduction

Assessing stress responses in natural populations has been of particular importance from a conservation perspective, as researchers attempt to evaluate the sub-lethal effects of anthropogenic activities on wildlife (Wikelski and Cooke, 2006; Arlettaz *et al*., 2007; Tarlow and Blumstein, 2007; Martin and Réale, 2008; Dantzer *et al*., 2014; Kersey and Dehnhard, 2014). Stress-induced effects on physiology and energetics may have significant consequences for individual health and reproductive success (Romero and Wikelski, 2001; Busch and Hayward, 2009), which ultimately impact population dynamics and viability (Boonstra and Singleton, 1993; Charbonnel *et al*., 2008). Stress responses can also be adaptive for healthy individuals, leading to enhanced survival in those individuals (Wikelski and Cooke, 2006).

The stress response in vertebrates, mediated in part by the hypothalamic–pituitary–adrenal (HPA) axis, involves the release of adrenocorticotrophic hormone (ACTH) into the circulatory system, which in turn acts on the adrenal cortex to produce glucocorticoids (reviewed by Tsigos and Chrousos, 2002). Diurnal variation in glucocorticoid concentrations follows a predictable circadian cycle (Son *et al*., 2011), and short-term elevation in response to acute stressors is part of daily life and necessary for an animal's survival in the wild. However, chronic activation of the HPA axis can lead to reproductive dysfunction, reduced fitness and disease (Boonstra *et al*., 1998; Wingfield and Sapolsky, 2003; but see Boonstra, 2013). Thus, the measurement of cortisol and/or its metabolites has been used to evaluate the degree of physiological stress an animal has experienced and gain an understanding of how environmental stressors affect adrenal function and cortisol production and, potentially, fitness.

Generally, cortisol levels have been assessed by sampling blood plasma or serum, saliva, urine or faeces. Ideally, the sample collection method should be non-invasive to minimize the stress associated with capture and handling of the animal (Delehanty and Boonstra, 2009). Given that each of these substrates reflects short-term physiological stress, ranging from minutes (plasma/serum) to several days (faeces), repeated sampling over time is required to obtain a complete temporal picture of physiological stress. Indeed, ecological studies of stress in wildlife have used faeces as a standard substrate for the measure of cortisol in part because of the non-invasive nature and ease of sample collection (e.g. Sheriff *et al*., 2011; Dantzer *et al*., 2014). Constraints on the use of faecal samples include the effects of diet and metabolic rate on metabolite levels found in the faeces, as well as a host of other concerns (Dantzer *et al*., 2011, 2014).

Recently, investigators have used hair to assess cortisol levels. Hair provides an attractive alternative for evaluating physiological stress for numerous reasons, as follows: (i) it is thought to incorporate blood-borne hormones during the growth phase of hair; (ii) it is relatively stable and can be stored and transported at room temperature; and (iii) any cortisol detected will reflect physiological stress experienced by the animal over the period of hair growth, often lasting weeks to months. In addition, although hair sampling may require capture or handling in certain cases (with the exception of using barbed wire at bait stations; Mowat and Strobeck, 2000; Ausband *et al*., 2011), any stress experienced during this event would not impact glucocorticoid levels in the collected hair. For these reasons, hair has been explored in a number of mammalian species, including reindeer/caribou (*Rangifer tarandus*; Ashley *et al*., 2011), grizzly bear (*Ursus arctos*; Macbeth *et al*., 2010) and rhesus monkey (*Macaca mulatta*; Dettmer *et al*., 2012), as an index of an animal's glucocorticoid production (hence, degree of physiological stress) over time.

Although hair cortisol concentration is increasingly being used for the assessment of physiological stress over long time periods in a number of contexts, there has been little effort to validate the physiological or biological relevance of hair cortisol values and determine whether they reflect cortisol levels in the blood accurately. Administration of exogenous ACTH can be used to determine the physiological relevance of cortisol extracted from samples other than blood. Increased circulating cortisol levels in response to the ACTH should be reflected in hair cortisol levels. In order for changes in cortisol concentrations to be detectable in the hair, a consistent and prolonged ACTH challenge (that is, repeated injections over time) must be administered, in comparison to the single injection that produces a measurable response in other substrates that do not take as long to accumulate cortisol or associated metabolites, such as blood, saliva, urine and faeces. In a recent study, Ashley *et al*. (2011) administered a single injection of ACTH to captive reindeer and caribou, and found that despite the detection of elevated cortisol levels in the faeces, no such response was observed in the hair. Terwissen *et al*. (2013) did successfully increase hair cortisol levels in Canada lynx (*Lynx canadensis*) using five weekly injections of ACTH but were not able to carry out any controls owing to the small number of animals available for the study. In contrast, Keckeis *et al*. (2012) found little uptake of cortisol in the hair of guinea pigs (*Cavia aperea f. porcellus*) after intraperitoneal injection with radioactive cortisol. Both Keckeis *et al*. (2012) and Cattet *et al*. (2014) conclude that hair follicles can produce cortisol, and thus hair cortisol concentrations should be interpreted cautiously.

In the present study, an ACTH challenge was carried out to validate the use of hair cortisol analysis in a marked natural population of eastern chipmunks (*Tamias striatus*). We expected elevated hair cortisol concentrations in ACTH-injected animals relative to saline-injected control chipmunks. We followed up this validation with a study of cortisol levels in eastern chipmunks in natural habitats and those habitats that had been recently logged.

Assessing the utility of hair cortisol in wildlife can also involve comparing results obtained from the novel source of cortisol (e.g. hair) with a previously validated source (e.g. faeces) when testing a hypothesis related to stress. Human activities, including habitat disturbance, can increase cortisol levels obtained from faeces in wildlife (Dantzer *et al*., 2014). Logging activities, in particular, disturb the forest ecosystem by disrupting habitat and therefore affecting nesting sites and food resources (Olsson and Staaf, 1995; Pinard and Putz, 1996; Leshyk *et al*., 2012), which can cause stress in wildlife (Suorsa *et al*., 2004; Banks *et al*., 2005; Martínez-Mota *et al*., 2007; Macbeth *et al*., 2010; Ashley *et al*., 2011; Leshyk *et al*., 2012). Habitat features in mature forests that are important for eastern chipmunks, such as the presence of large trees at low density with limited low-level shrubs (Svendsen and Yahner, 1979), are altered by various silviculture techniques (Olsson and Staaf, 1995; Doyon *et al*., 2005). Using measures of cortisol from both faeces and hair, we tested the prediction that eastern chipmunks from areas logged via single-tree selection would be more stressed (have higher cortisol levels) than chipmunks from natural sites. We also expected that the results would be concordant between faeces and hair.

## Materials and methods

The Animal Care Committee at Laurentian University approved all procedures prior to implementation in both the wild and captive studies. All chemicals were obtained from Sigma-Aldrich Canada Ltd (Oakville, ON, Canada) unless otherwise specified.

### Hair cortisol validation

An ACTH challenge was conducted on a wild population of eastern chipmunks in Algonquin Provincial Park, Ontario, Canada (45**°**30′N, 78**°**40′W) from May to August of 2011. Longworth traps were placed 15 m apart in a 2100 m^2^ grid composed of Longworth traps. Traps were baited in the morning with water-soaked sunflower seeds and checked twice with a 2 h interval in between. Traps were set 5 days per week for an initial period to maximize the number of individuals initiated into the experimental treatments. Only adults were used in our experiment. Upon capture, each chipmunk was examined for sex, marked with two ear tags (model 1005-1; National Band and Tag, Newport, KY, USA) and weighed with a Pesola scale. Callipers were used to measure skull length (distance from occipital crest to tip of nose; ±0.1 mm) and skull width (zygomatic breadth; ±0.1 mm), while a ruler was used to measure the right hind-foot length from the heel to the tip of the longest nail (±1 mm). Each chipmunk had a 2 cm × 2 cm patch of hair shaved from the right hindlimb using an electric razor (ConAir Beard and Moustache Trimmer model GMT100RQCS, Stamford, CT, USA) at the start of the study.

Chipmunks were assigned to a treatment upon first capture in an alternate design, i.e. the first chipmunk was assigned the ACTH injection (see below), and the second chipmunk was assigned as a control (see below). This pattern of assignment continued until we had captured as many chipmunks as possible. The protocol used for injections was modified from that of Bosson *et al*. (2009). Chipmunks were injected with 0.2 ml of either Synacthen Depot (CDMV Inc., St Hyacinthe, QC, Canada) or saline solution (control) in the caudal thigh muscle. The Synacthen Depot solution was mixed immediately prior to entering the study site by adding 235 µl of sterile saline to 15 µl of Synacthen Depot in a vial to give 10 IU/kg based on an average 150 g body weight. The solution was kept cold in a cooler bag with freezer packs until used. All injections were given using a tuberculin syringe (1 ml syringe, 27 gauge, ½″ needle; CDMV Inc.). Injections occurred between and 09.00 and 12.00 h, and began in late May and ended in early August.

After the initial injection of the chipmunks, the animals were trapped on a weekly basis in order to continue to inject individual chipmunks with ACTH or saline (depending on their assigned treatment). Weekly trapping and injections of the chipmunks continued until the hair on the shaved patch grew back to a length similar to that of the original hair sample. The new hair was shaved from the site of the initial shave (right hindlimb), placed in a labelled 1.5 ml Eppendorf tube and stored at room temperature.

#### Hair extraction and cortisol analysis

Hair samples were delivered to the Endocrinology Laboratory at the Toronto Zoo for analysis. The hair was cut into 5 mm pieces and placed into a 7 ml glass scintillation vial to be weighed. To avoid contamination with other biological fluids that may have artificially elevated cortisol levels, all hair samples were washed with 100% methanol by vortexing in a tube for 10 s and immediately removing all of the 100% methanol using a pipettor. Immediately thereafter, 80% methanol in water (v:v) was added to the samples, at a ratio of 0.001–0.005 g/ml, and samples were vortexed for 5–10 s. Samples were then mixed for 24 h on a plate shaker (MBI Orbital Shaker; Montreal Biotechnologies Inc., Montreal, QC, Canada). After 24 h, the vials were centrifuged for 10 min at 2400*g*. The supernatants were pipetted off into clean glass vials and dried down under air in a fume hood. The dried extracts were stored at −20°C until analysis.

Samples were removed from the freezer and brought to room temperature on the laboratory bench prior to analysis. Reconstitution of the dried-down extracts was done by adding assay buffer (from Cortisol EIA Kit, no. K003-H1; Arbor Assays, Ann Arbor, MI, USA) and sonicating for 20 s followed by vortexing for 10 s. Final volumes of assay buffer added to each sample resulted in chipmunk extracts being neat. All samples were centrifuged for 1 min at 1200*g* immediately prior to dispensing onto the microtitre plate. Cortisol concentrations in the reconstituted extracts were assayed following the manufacturer's instructions for the Cortisol EIA Kit. The detection limit of the assay was 45.4 pg/ml as per manufacturer's specifications. Results are presented as nanograms of cortisol per gram of hair.

The efficiency of the hair extraction procedure was analysed through recovery of exogenous cortisol added to chipmunk hair samples prior to extraction. Briefly, 10 samples of at least 0.001 g each from a pool of chipmunk hair were used; five samples were spiked with 5 µl of 52 pg/µl cortisol in 100% methanol, and five samples were left unspiked. The samples were mixed and then extracted and assayed as described above. The percentage efficiency was calculated using the following formula: amount observed/amount expected × 100%, where the amount observed is the value obtained from the spiked sample minus background and the amount expected is the calculated amount added. The percentage efficiency is presented as the mean ± SEM.

Assay validation was carried out as follows. First, parallel displacement between the standard curve and a serial dilution of a pooled chipmunk hair extract was used to detect immunological similarities between the standard and sample hormones. The resulting curves were plotted as relative dose vs. percentage antibody bound, and linear regression analysis was performed. Sample dilution was selected based on 50% binding of the pooled sample curve. Second, possible interference of components within the extract with antibody binding was analysed through recovery of exogenous cortisol added to pooled chipmunk hair extracts. Briefly, 75 µl aliquots of extract from a pooled chipmunk hair sample were spiked with 75 µl of each hormone standard. The samples were assayed as described above. The percentage recovery was calculated using the following formula: amount observed/amount expected × 100%, where the amount observed is the value obtained from the spiked sample and the amount expected is the calculated amount of standard hormone added plus the amount of endogenous hormone in the unspiked sample. The percentage recovery is presented as the mean ± SEM. The graph was plotted as hormone added vs. hormone recovered, and regression analysis was used to determine whether there was a significant relationship between them. Third, for both species, all hair samples were processed, extracted and analysed at the same time in an attempt to minimize the effects of possible variation associated with day-to-day differences in extraction and assay efficiency. All samples were assayed in duplicate, with the mean of the two results being presented. Only duplicates with <10% coefficients of variation (CVs) were accepted as data; an average of 5.2% intra-assay CV was observed across all duplicates. The inter-assay CV, based on controls of laboratory stocks of pooled faecal extracts obtained from female spotted-necked otters (*Hydrictis maculicollis*) run at 65% binding, was 4.5%.

#### Statistical analysis

We conducted one-way analysis of variance to determine whether there were differences in hair cortisol concentration between ACTH and saline treatments, both before and after the ACTH challenge (statistica 10; Statsoft, Inc., Tulsa, OK, USA). In addition, we used Student's paired *t*-tests to compare the hair cortisol concentration of chipmunks before and after each of the ACTH challenge or saline injections. Unless noted, all data were normal (Shapiro–Wilks test, *P* > 0.1). All tests were two tailed, with type 3 sum of squares.

### Field study of logged and natural sites

#### Site

Field research was conducted from May to August 2012 in Algonquin Provincial Park, Ontario, Canada. The Algonquin Forest Authority oversees a number of different types of logging practices in Algonquin Park, including selection cutting, which removes 30% of the canopy cover (Ontario Ministry of Natural Resources, 1998). Trapping grids were set up in two natural sites that had not been harvested for at least 72 years and four logged sites that had been harvested by single-tree selection (a type of selection logging) within the last 5 years (i.e. they were not being logged at the time of the study; J. Yaraskavitch and R. Tozer, Ontario Ministry of Natural Resources, personal communication, November 2012). All sites were hardwood mixed-wood forests dominated by fir (*Abies* spp.) and spruce (*Picea* spp.; Ontario Ministry of Natural Resources, 1997).

Each grid began 10–20 m from the road, and traps were placed between 8 and 15 m apart (Bowman *et al*., 2001). All the grids, except for two of the logged grids, were evenly distributed in a rectangular pattern. Two logged sites were situated along the edge of a logged area, and thus the grids were irregularly shaped. No traps were placed outside the logging zones. GPS points were taken of select traps around the perimeter of each grid to measure the grid area.

#### Field procedures

Field procedures followed those noted above unless stated otherwise. Hair samples were collected as described above except that a 1 cm × 1 cm patch of hair was shaved from the thigh of the right hindlimb. A maximum of three faecal samples per chipmunk of >0.025 g were collected with at least 72 h between collections (see Dantzer *et al*., 2010). Chipmunks were fitted with ear tags, and data for weight, sex, age, hind-foot length and skull width and length were collected for all adult chipmunks.

Faecal samples were weighed to an accuracy of ±0.001 g and transferred into 1.5 ml Eppendorf tubes. Into each tube, 80% methanol in distilled water (v:v) was added at a ratio of 0.100 g of faeces per 1 ml of methanol solution. Samples were kept refrigerated until analysis. Both hair and faecal samples were processed at the Endocrinology Laboratory at the Toronto Zoo in December 2012.

#### Sample extraction and cortisol analysis

##### Hair

Hair samples were processed as noted above (see ‘*Hair extraction and cortisol analysis*’).

##### Faeces

Immediately prior to extraction, the faecal pellets within each Eppendorf tube were broken up with a clean spatula and mixed with the methanol. The samples were vortexed briefly and then mixed overnight on a plate shaker (Barstead Lab-Line Multi-Purpose Rotator; VWR, Mississauga, ON, Canada). Each sample was centrifuged for 10 min at 2400*g*, and the methanol extract (the supernatant) was transferred to a new 7 ml glass scintillation vial. The extracts were stored at −20°C until analysis.

##### Cortisol enzyme immunoassay

Samples were removed from the freezer and brought to room temperature on the laboratory bench prior to analysis. Dried hair extracts were reconstituted to the original extract volume (neat) by adding assay buffer (0.1 mm sodium phosphate buffer, pH 7.0, containing 9 g of NaCl and 1 g of bovine serum albumin per litre) and sonicating for 20 seconds followed by vortexing for 10 s. Faecal extracts were diluted 1:50 in assay buffer and vortexed for 10 s.

Faecal cortisol analysis was previously validated in eastern chipmunks by Montiglio *et al*. (2012). Hair cortisol and faecal cortisol metabolites were quantified using a method modified from Munro and Lasley (1988). Cortisol antiserum (R4972; C. Munro, University of California, Davis, CA, USA) was diluted in coating buffer (50 mm bicarbonate buffer, pH 9.6) at 1:12 000. The cross-reactivities of the cortisol antiserum were as follows: cortisol, 100%; prednisolone, 9.9%; prednisone, 6.3%; cortisone, 5%; corticosterone, 0.7%; 21-deoxycortisone, 0.5%; deoxycorticosterone, 0.3%; and other, <0.3%. Cortisol–horseradish peroxidase conjugate (C. Munro, University of California, Davis, CA, USA) was diluted in assay buffer at 1:60 000. The standard used was cortisol (Steraloids Inc., Newport, RI, USA; catalogue no. Sigma H-0135; 0.078–20 ng/ml). Only duplicates with <10% CVs were accepted as data; an average of 3.6% intra-assay CV was observed. The inter-assay CV, based on controls of laboratory stocks of pooled faecal extracts obtained from female spotted-necked otters (*H. maculicollis*) run at 65% binding, was 7.7%.

Microtitre plates (Nunc Maxisop; VWR, Mississauga, ON, Canada) were coated with 50 μl of cortisol antibody diluted in coating buffer and incubated overnight at 4°C. Unbound antiserum was washed from coated plates with 0.02% Tween 20 solution three times using a microplate washer (Bio-Tek Instruments, Winooski, VT, USA). Following the wash, 50 μl of hair or faecal samples, standards and controls diluted in assay buffer were added to wells in duplicate, followed by 50 μl of cortisol–horseradish peroxidase conjugate diluted in assay buffer. Plates were incubated for 2 h at room temperature. Following incubation, the plates were washed three times, and 100 μl of substrate solution (50 mm citrate, 1.6 mm hydrogen peroxide and 0.4 mm 2,2′-azino-di-(3-ethylbenzthiazoline sulfonic acid) diammonium salt, pH 4.0) was added. Absorbance was measured at 405 nm using a spectrophotometer (MRX microplate reader, Dynex Technologies, Chantilly, VA, USA) 30–45 min after the substrate was added. Results are presented as nanograms of cortisol per gram of hair or wet faeces.

#### Statistical analysis

The population density of each grid was calculated using the spatially explicit capture–recapture (secr) package version 2.4.0 in R version 2.15.1 (R Core Team, 2012; Efford, 2013). A spatial model and the probability of capturing an animal at a certain distance from the centre of its home range were used to estimate the population density in the area of each trapping grid. A point shapefile (spatial model) was created, using Geographic Information Software (Esri, Redlands, CA, USA), representing GPS points of each trap in each grid. The population density was calculated using Maximum Likelihood estimator (Efford, 2004), which assumes that an animal is most likely to be captured at the centre of its home range. The default buffer of 100 m around each grid was used. The model with the best fit incorporates a learned response in the group of chipmunks and a trend over time (Efford, 2004; Borchers, 2010).

Three body dimension measurements were taken for each chipmunk (skull length, skull width and hind-foot length). These dimensions were not independent, so a principal components analysis was applied to create a linear combination of the average body dimension measurements for each chipmunk. A regression of the first principal component on mass was performed to obtain residuals as an index of body condition for each chipmunk, except pregnant females (*n* = 4; Schulte-Hostedde *et al*., 2005).

Two faecal samples were removed as outliers because their cortisol concentrations were >3 SD from the mean. The faecal samples had repeated measures, so an average was taken per chipmunk after the outliers were removed for analyses that involved comparisons between hair and faecal cortisol. Faecal cortisol metabolite concentrations were natural-log transformed to meet normality assumptions. A linear mixed-effect model was run to test the null hypothesis that there is no difference between faecal cortisol metabolite concentrations of the chipmunks from logged or natural sites using the lme4 (Bates *et al*., 2013) in the R environment (R version 2.15.1; R Core Team, 2012). The independent variables were sex, age (adult or juvenile), treatment (logged vs. natural), density (chipmunks per hectare) and body condition index. Both population density and body condition have been related to stress in wildlife (Rogovin *et al*., 2003; Raouf *et al*., 2006; Waye and Mason, 2008; Williams *et al*., 2008). Significance of the fixed effects was determined by bootstrapped likelihood confidence intervals. The repeatability of cortisol among individuals was calculated from variance components from the model (Lessells and Boag, 1987).

Individual variables were assessed for normality using histograms. One hair cortisol concentration was >6 SD from the mean cortisol concentration for all the chipmunks, so was removed as an outlier. Otherwise, data were log transformed when necessary to maintain normality assumptions. A linear model was run to test the null hypothesis that there is no difference in hair cortisol concentrations of the chipmunks from logged or natural sites. The independent variables were sex, age (adult or juvenile), treatment (logged vs. natural), density (chipmunks per hectare) and body condition index. General model assumptions were checked for all models by plotting residuals, as well as Q-Q and leverage plots. All tests were two tailed, with type 3 sum of squares.

## Results

### Hair cortisol validation

**Figure 1:**
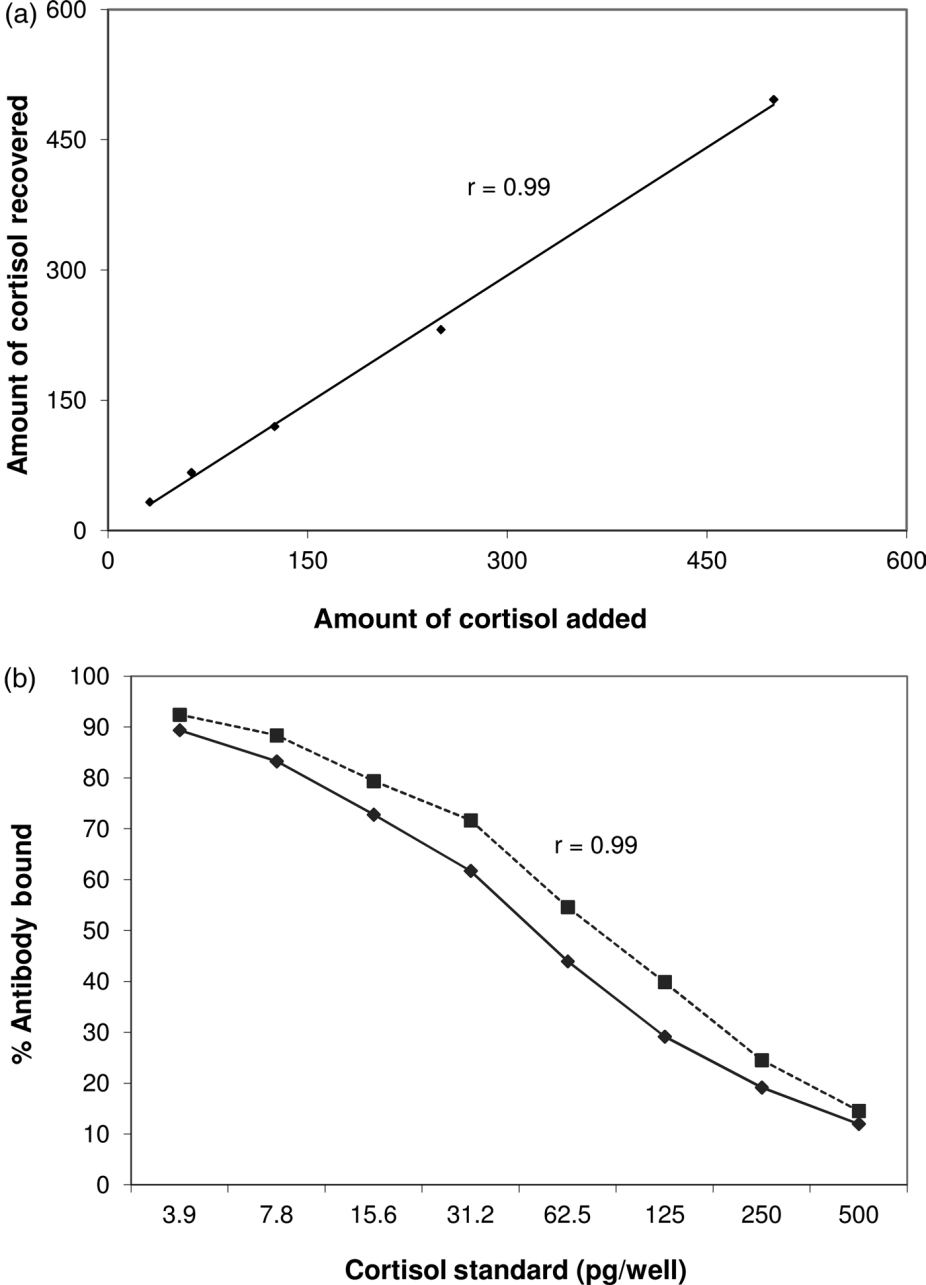
Cortisol assay validation. (**a**) Recovery of exogenous cortisol from pooled hair extracts. (**b**) Parallelism for serial dilutions of pooled hair extracts against the cortisol standard curve. Symbol key: filled diamonds, standard curve; and filled squares, pooled hair extract dilutions.

Extraction of exogenous cortisol in chipmunk hair samples resulted in procedure efficiency of 102.5 ± 5.6%. The recovery of known concentrations of cortisol from chipmunk hair extracts was 100.1 ± 2.8%. The measured hormone concentrations in the spiked samples correlated with the expected concentrations (*r* = 0.99, *P* < 0.01; Fig. 1a). Serial dilutions of pooled hair extract showed parallel displacement with the cortisol standard curve (*r* = 0.99) at *P* < 0.01 (Fig. 1b).

Initial capture of chipmunks led to *n* = 23 individuals that were sampled, including hair collection. The mean (±SD) mass of chipmunks injected with ACTH was 90.0 ± 8.80 g, whereas chipmunks injected with saline had a mean mass of 87.4 ± 12.15 g on initial capture. Subsequent captures and injections occurred over the course of 8–9 weeks, starting in late May and early June. Not all chipmunks were recaptured and injected weekly (many disappeared from our grid), thus the final number of chipmunks that received weekly injections until the final hair sample was obtained differed from the number initially captured. Final hair samples were collected from *n* = 12 eastern chipmunks (*n* = 5 injected with ACTH and *n* = 7 injected with saline). Saline-injected chipmunks were injected a mean of 5.71 times (SD = 3.04) over a mean of 54.86 days (SD = 20.84), whereas ACTH-injected chipmunks were injected a mean of 5.40 times (SD = 1.82) over 64.2 days (SD = 13.77). There were no differences between ACTH and saline-injected chipmunks in terms of the number of injections (*t* = 0.2, d.f. = 10, *P* = 0.84) or the number of days over which the injections were made (*t* = 0.87, d.f. = 10, *P* = 0.4). We calculated the number of days between the final injection and the final shave of hair from the chipmunks, and found no significant difference between chipmunks injected with ACTH and saline (12.6 days vs. 23.0 days; *t*-test assuming unequal variances, *t* = 0.935, *P* = 0.39). One chipmunk from the saline group had its hair sample shaved 64 days after its last saline injection, but otherwise the time between the last injection and the final hair sample ranged from 6 to 26 days.

**Figure 2:**
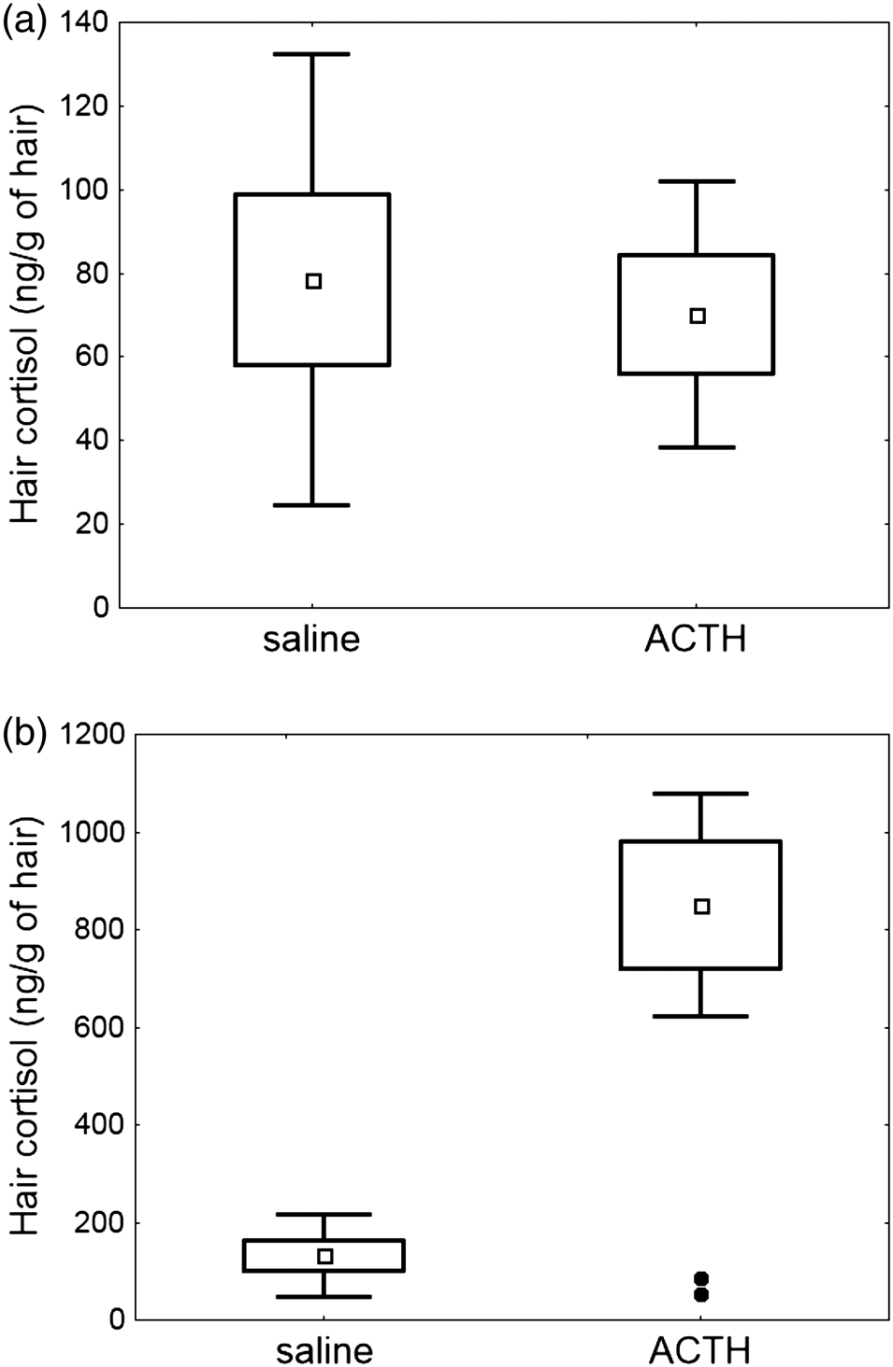
Hair cortisol concentrations of saline- and adrenocorticotrophic hormone (ACTH)-injected eastern chipmunks. (**a**) Initial hair cortisol concentrations were similar for animals in both control (saline) and ACTH treatment groups (*P* = 0.76). (**b**) The ACTH-injected chipmunks had significantly higher hair cortisol concentrations than control chipmunks (*P* < 0.001). The mean, standard error (box) and standard deviation (whiskers) are presented. Data points from the ACTH treatment represent two chipmunks that did not respond to the ACTH injection.

Cortisol levels from initial hair samples showed no significant differences between chipmunks designated to the ACTH and saline treatments (*t* = 0.31, d.f. = 10, *P* = 0.76; Fig. 2a). Cortisol levels from regrown hair samples were significantly different between ACTH-treated and saline-treated chipmunks (*F* = 5.46, d.f. = 10, *P* = 0.042), but variances were significantly different between the two groups (*F*-ratio test = 29.11, *P* < 0.001). Inspection of the data indicated that two chipmunks from the ACTH-injected group did not respond in terms of hair cortisol concentration. Hair cortisol concentrations for the three eastern chipmunks that did respond ranged from 603.72 to 1050.04 ng/g of hair, whereas the two individuals that did not respond to the ACTH treatment had hair cortisol concentrations of 52.53 and 86.24 ng/g of hair (cortisol concentrations from the initial shaved hair sample for these chipmunks were 75.61 and 56.51 ng/g of hair, respectively). These latter two values were within the range of hair cortisol levels from saline-injected controls (40.27–260.22 ng/g of hair). Removal of the non-responding chipmunks led to a significant difference in hair cortisol concentration (*F* = 59.33, d.f. = 8, *P* < 0.001; Fig. 2b) and homogeneity of variance between the two groups (*F*-ratio = 7.17, *P* > 0.05; Fig. 2b), with ACTH-injected chipmunks having 542.2% higher hair cortisol concentration than saline-injected chipmunks. Dependent *t*-tests of hair cortisol concentrations before and after saline injections were not significantly different (*t* = 1.61, d.f. = 6, *P* = 0.16), whereas hair cortisol concentration was significantly higher after ACTH challenge following removal of the two non-responders (*t* = 6.33, d.f. = 2, *P* = 0.024).

The lack of response to the ACTH injections was investigated, but no evidence was found indicating that the number of injections (*r* = 0.22, *P* = 0.72) or the number of days between the first and last injection (*r* = 0.33, *P* = 0.58) were related to hair cortisol concentrations in chipmunks injected with ACTH. Other factors typically associated with lack of response following ACTH challenge include low dosage of exogenous ACTH or adrenal insufficiency due to illness or reduced health resulting from chronic stress. Examination of the body mass of both individuals showed that one individual that did not respond to ACTH injection was significantly heavier (103.7 g) than the remaining chipmunks injected with ACTH (mean = 87.06 g, SD = 3.83; one-sample *t*-test, *t* = 8.65, d.f. = 3, *P* = 0.003). An assessment of body condition was carried out on all chipmunks in the study (residual mass; Schulte-Hostedde *et al*., 2005), using hind-foot length as an index of body size (body mass–hind-foot length regression, *r*^2^ = 0.44, *P* = 0.019). The second chipmunk that did not respond to the ACTH injection had a body condition (−0.063) that was significantly lower than the remaining chipmunks that were injected with ACTH (mean = 0.008, SD = 0.01; one-sample *t*-test, *t* = 7.35, d.f. = 3, *P* = 0.005).

### Field study of logged and natural sites

The estimated population density (95% confidence intervals in parentheses) of chipmunks in the natural sites was 4.48 (0.56–35.42) and 18.60 (8.94–38.72) chipmunks/ha, whereas the population density of chipmunks in logged sites was 3.18 (1.35–7.42), 4.87 (2.03–11.66) and 14.00 (4.85–40.43) chipmunks/ha.

The first principal component from the principal components analysis explained 54% of the variance in the three body dimension variables. Hind-foot length (0.43), skull length (0.67) and skull width (0.60) loaded heavily and positively on the first principal component. Thus, individual scores from the first principal component were used as an index of body size of each chipmunk (Schulte-Hostedde *et al*., 2001).

**Figure 3:**
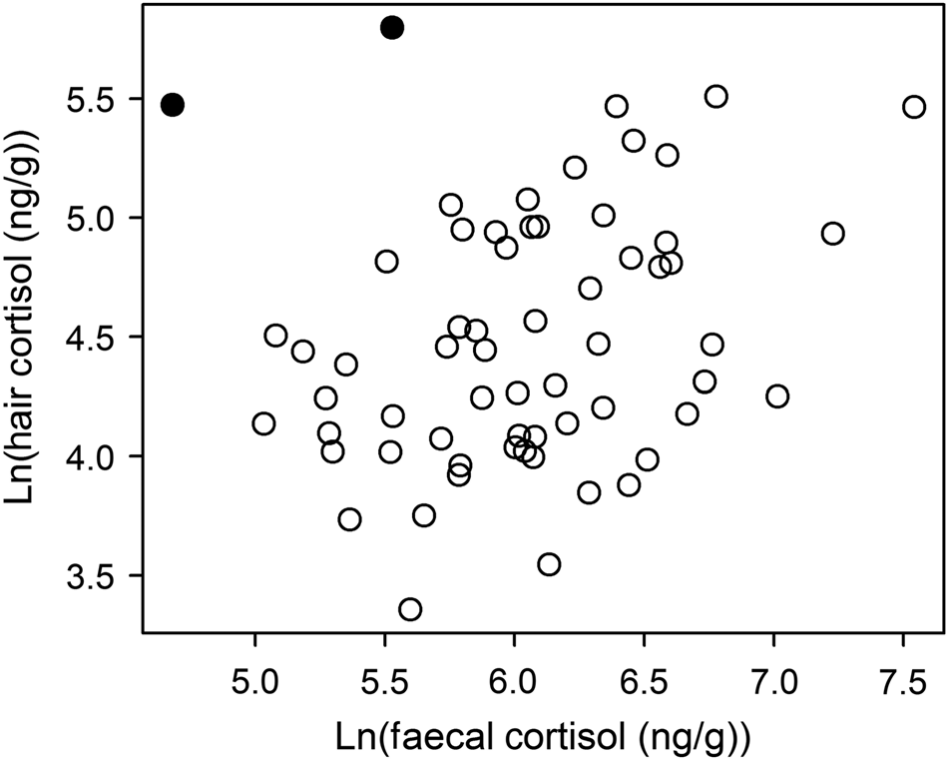
Hair and average faecal cortisol levels (expressed as nanograms per gram of substrate) were positively correlated in wild-caught chipmunks. The filled circles highlight two individuals with high levels of hair cortisol but low levels of faecal cortisol. Results are presented in the text with these points included (*P* = 0.055) and excluded (*P* < 0.001). Cortisol levels were natural-log transformed.

The number of faecal samples collected per individual with a minimum of 72 h between samples had a range of two to four (mean = 3.06 ± 0.84 SD). Levels of cortisol and its metabolites from hair and faeces were positively correlated (*n* = 62, *r* = 0.25, *P* = 0.055; Fig. 3) among wild chipmunks. In two individuals, there was a notable discordance between hair and faecal cortisol levels, but with these two individuals removed the two measures were strongly related (*n* = 60, *r* = 0.41, *P* < 0.001). Thus, individuals with high hair cortisol levels also had high levels of faecal cortisol metabolites.

**Figure 4:**
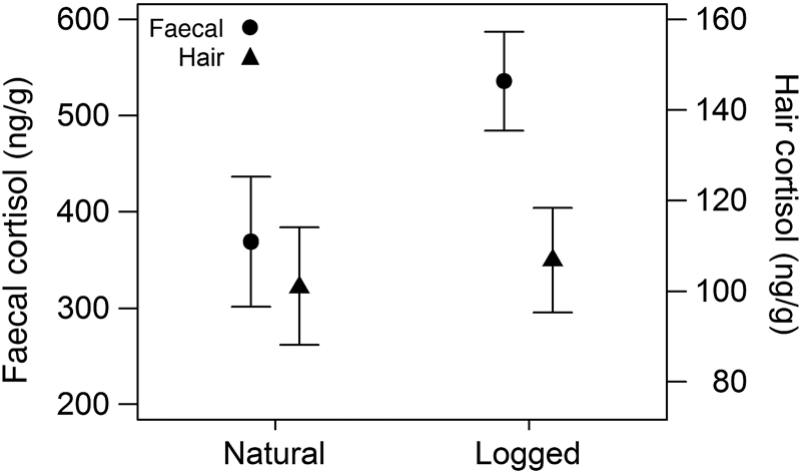
Chipmunks caught in natural sites had lower levels of faecal cortisol (expressed as nanograms per gram of faeces; mean ± SEM) than those trapped in logged sites. The non-transformed data are shown, with average faecal values plotted on the left and hair values on the right.

Faecal cortisol levels had a moderately high level of repeatability (*r* = 0.67). Chipmunks from logged sites had higher faecal cortisol metabolite levels than those from natural sites (Table 1 and Fig. 4). Individuals trapped in sites of higher density also had higher faecal cortisol metabolite levels, and there was tendency for individuals that were in better condition to have higher faecal cortisol metabolite levels as well (Table 1). Chipmunks from natural and logged sites did not differ in body condition (*t*_1,66_ = −0.90, *P* = 0.37). Similar relationships were not observed for hair cortisol levels. None of the effects was significant when using hair cortisol as the dependent variable (Table 1). To emphasize further that faecal and hair cortisol resolve different relationships, when hair cortisol was included as a term in a linear model with average faecal cortisol model as the dependent variable, the effects of all terms remained similar, including the treatment effect (*t* = 2.47, *P* = 0.02), despite the strong positive relationship between average faecal and hair cortisol levels within individuals.

**Table 1:** Influence of environmental factors and individual traits on faecal and hair cortisol levels in wild chipmunks

Parameter	Effect	Estimate	SEM	*t*	*P* Value	Lower CI	Upper CI
Faecal cortisol	Treatment	0.512	0.157	3.27	–	**0.2166**	**0.8072**
	Condition	0.017	0.009	1.80	–	−0.0008	0.0344
	Sex	−0.101	0.139	0.73	–	−0.3628	0.1608
	Age	0.018	0.185	0.10	–	−0.3298	0.3673
	Density	0.034	0.013	2.59	–	**0.0093**	**0.0590**
Hair cortisol	Treatment	0.057	0.166	0.35	0.73	–	–
	Condition	0.003	0.011	0.32	0.75	–	–
	Sex	0.196	0.157	1.25	0.22	–	–
	Age	0.194	0.208	0.93	0.36	–	–
	Density	−0.005	0.014	−0.33	0.74	–	–

Chipmunks were caught in either natural or logged sites (treatment effect) that varied in density. Condition, sex and age are attributes of individuals. For the fixed effects in the faecal (linear mixed-effects) model, we report bootstrapped confidence intervals (CIs) as tests of the effects. Significant effects (bold) are those that do not cross zero. Standard *P* values are given for the hair cortisol linear model.

## Discussion

The ACTH challenge of a wild population of eastern chipmunks resulted in significantly higher hair cortisol concentrations than those in the control animals (saline injection). This is the first report validating the use of hair cortisol for studies of physiological stress in a rodent species, and one of only three reports characterizing changes in hair cortisol concentration following ACTH injection in mammals (cattle, González-de-la-Vara *et al*., 2011; lynx, Terwissen *et al*., 2013), underlining the value of this technique in assessing the impact of both natural and anthropogenic effects on the physiological state of free-ranging and captive wildlife populations. Furthermore, these results highlight the need for depot or slow-release formulations of ACTH with repeated injections over the period of hair growth to detect significant changes in hair cortisol levels.

Our results identified two eastern chipmunks labelled as non-responders because no concomitant increase in hair cortisol concentrations was observed. Potential mitigating factors include under-dosage with ACTH in one animal with a significantly higher body mass and compromised health in the other animal as suggested by significantly reduced body condition. Evidence that poor body condition or exposure to negative environments affects HPA axis function has been shown in various species. Rats subjected to bacterial sepsis following experimentally induced caecal ligation and puncture had lower adrenal corticosterone levels compared with sham-operated control animals (Koo *et al*., 2001).

We also used eastern chipmunks to examine the utility of hair cortisol in natural populations by testing the hypothesis that logging practices enhance cortisol levels (Suorsa *et al*., 2004; Banks *et al*., 2005; Martínez-Mota *et al*., 2007; Macbeth *et al*., 2010; Ashley *et al*., 2011; Leshyk *et al*., 2012) as a result of habitat disturbance. We expected single-tree selection logging to disrupt the landscape sufficiently to enhance stress in eastern chipmunks and to increase cortisol levels in these chipmunks relative to those captured on undisturbed sites. Our results were inconsistent with our initial prediction and depended on which substrate was used in terms of cortisol levels. On the one hand, faecal cortisol metabolites were significantly higher in chipmunks captured in logged sites than in undisturbed sites, but on the other hand, hair cortisol did not differ between these two groups. These results might best be understood in the context of the temporal and spatial scale of our study.

Cortisol is deposited in hair over a lengthy period during the moult and, as such, hair cortisol measures represent stress over the longer term on the scale of weeks and months, whereas faecal cortisol metabolites provide a stress profile that represents the most recent hours or days. Samples from logged sites were taken from the periphery, and sampled chipmunks can move from logged to undisturbed habitat readily. Thus, it appears that while chipmunks captured on logged sites had greater short-term stress than chipmunks captured on undisturbed sites, stress over the long term was not significantly different between them, perhaps because of the likelihood that chipmunks are not spending all of their time in logged habitat patches. Indeed, if logged habitat patches are stressful to chipmunks, they may actively avoid these areas (Ford and Fahrig, 2008), leading to the patterns we have observed. In contrast, because faecal cortisol metabolites reflect stress experienced over the preceding hours to days, this measure may be more sensitive to differences in cortisol at the temporal scale of our study.

The results of logging activities include an altered landscape and degraded habitat quality that can be stressful for wildlife (Busch and Hayward, 2009). For example, various measures of stress in breeding birds have been positively associated with forest harvesting (Suorsa *et al*., 2003; Lucas *et al*., 2006). This is consistent with the general trend that human activities cause increases in faecal glucocorticoids in wildlife (Dantzer *et al*., 2014). The mechanism behind the effects of logging on cortisol production are unclear; it is unknown what aspect of affected habitat quality is stressful to wildlife, and why. Future research should examine this important question because it may allow for the mitigation of sub-lethal effects of logging, such as enhanced stress on wildlife.

The data obtained in this study support the use of hair cortisol analysis as a method of evaluating stress over long time scales in wild populations, thereby providing valuable information on the population response to changes in the environment. The benefits of using hair as a substrate for cortisol analysis in comparison to traditional samples (e.g. faeces, urine, blood) include the following: stability of the sample; lack of influence of collection/storage technique; use of non-invasive sampling techniques (e.g. barbed wire); and, most importantly, a measurement of stress over time without the confounding effects of daily or acute changes in cortisol response. Nonetheless, it is important to remember that mammals engage in a moult, which can occur seasonally, beginning and ending at specific points in time (progressional moult) or growing continuously (diffuse moult; Sare *et al*., 2005). Cortisol levels from hair samples reflect physiological stress during the period of hair growth, which may or may not immediately precede the sampling period. Thus, investigators should take moulting patterns into account when interpreting cortisol concentrations from hair in order to be certain about the temporal period reflected in the cortisol profile obtained.

While the benefits of a stable, non-invasive substrate for cortisol (or other glucocorticoids) have led to the increasing use of hair as a substrate with which to evaluate long-term cortisol levels (e.g. Macbeth *et al*., 2010; Bryan *et al*., 2014), the validation of hair has not been so straightforward. The validation of hair as an appropriate substrate for cortisol measurements has been complicated by a lack of controls (Terwissen *et al*., 2013) and a protocol that involved a single injection of ACTH (Ashley *et al*., 2011) rather than a series of ACTH injections with a slow-release formula of ACTH (present study). Other studies have found little uptake of cortisol by hair (e.g. Keckeis *et al*., 2012; Cattet *et al*., 2014), challenging the assumption that hair cortisol levels are the result of cortisol diffusing from the blood supply to the hair follicles. As suggested by Keckeis *et al*. (2012) and Cattet *et al*. (2014), a study by Ito *et al*. (2005) concluded that human hair follicles have local HPA-like activity capable of stimulating the secretion of cortisol in response to corticotrophin-releasing hormone. Therefore, there is the possibility that hair cortisol levels are the result of both systemic and local cortisol production, and thus, the concern that hair cortisol levels may reflect an acute stress response due to the follicle's HPA-like function. However, lack of increase in hair cortisol concentrations in the control (saline-injected) chipmunks in the present study indicates that the acute stress resulting from trapping, handling and injecting the chipmunks did not impact hair cortisol levels. Clearly, the mechanism of cortisol deposition in hair needs to be studied further in order to reconcile these concerns.

We also showed that hair cortisol is correlated with faecal cortisol metabolites in eastern chipmunks and that while chipmunks from logged habitat had higher faecal cortisol metabolites than those from undisturbed habitat, hair cortisol did not detect these differences. Although some further studies are still required to understand better the principles of cortisol deposition in hair and the effects of hair texture/density, colour and location on cortisol levels, the present study provides the necessary foundation for validating hair cortisol analysis in wild populations. We do caution the use of hair cortisol for detecting differences in physiological stress when comparing individuals within populations and suggest that it is best suited to the examination of population-level differences.

## Funding

This work was supported by the Canada Research Chair in Applied Evolutionary Ecology; Natural Sciences and Engineering Research Council of Canada Discovery Grant and Undergraduate Student Researcher Award; and Canadian Foundation for Innovation Leaders Opportunity Fund to A.I.S.-H., and by the Toronto Zoo Endangered Species Reserve Fund to G.F.M.
